# Beyond Anxiety and Depression: Multidimensional Psychiatric Screening for Neurological Patients Based on HADS and BSI-53

**DOI:** 10.2147/NDT.S503053

**Published:** 2025-04-26

**Authors:** Milenko Kujovic, Daniel Benz, Bruno Baumann, Christina Engelke, Zsofia Margittai, Julia Christl, Rüdiger J Seitz, Til Menge, Christian Bahr, Mathias Riesbeck, Eva Meisenzahl, Christian Schmidt-Kraepelin

**Affiliations:** 1 Department of Psychiatry and Psychotherapy, Medical Faculty, Heinrich-Heine-University Düsseldorf, Düsseldorf, Germany; 2 Centre for Neurology and Neuropsychiatry, LVR-Klinikum Düsseldorf, Medical Faculty, Heinrich-Heine-University Düsseldorf, Düsseldorf, Germany; 3 Department of Psychiatry, University Hospital Münster, University of Münster, Münster, Germany; 4 Department of Psychiatry and Psychotherapy, Kaiserswerther Diakonie, Florence Nightingale Hospital, Düsseldorf, Germany

**Keywords:** psychiatry, neurology, BSI-53, HADS-D, depression, anxiety

## Abstract

**Objective:**

Although neurological patients are known to suffer from psychiatric comorbidities, appropriate, cost-effective, standardized screening measures are still scarce in clinical practice. We examined a multidimensional psychiatric screening for neurological patients.

**Methods:**

We report a retrospective analysis of multidimensional psychiatric screening using the HADS-D and the BSI-53 in 437 consecutive neurological inpatients. The HADS-D describes depressive and anxiety symptoms, while the BSI-53 expands the symptom clusters associated with psychiatric disorders by somatization, obsessive-compulsion, interpersonal sensitivity, hostility, paranoid ideation, psychoticism and global measures of mental distress. Patients were separated in diagnostic groups (vascular, demyelinating, degenerative, epileptic, other) with regard to their diagnosis.

**Results:**

Our results corroborate previous findings of high prevalence of psychiatric symptoms particularly in younger patients (< 60 years). Furthermore, 27.2% were above the HADS-D cut-off for anxiety and 25.4% for depression. We found no differences between diagnostic groups. Importantly, we also show that neurological patients suffer from mental distress beyond anxiety and depression, around 16–30% depending on age and gender.

**Conclusion:**

We have shown that a notable proportion of neurological patients report psychiatric symptoms, thus emphasizing the importance of a thorough, multidimensional psychiatric screening in neurological patients.

**Trial Registration Number:**

DRKS00030528, date of registration: 2022-11-04 retrospectively registered.

## Background

The widespread prevalence of psychiatric comorbidity in neurological patients is well substantiated by research findings.^1^ However, there is evidence of their underestimation in clinical practice internationally.^2^ As a result, multidisciplinary approaches^3^ are scarce in clinical treatment, even though a lack of appropriate, joint management of psychiatric and neurological symptoms is known to lead to adverse outcomes.^4^ For example, psychiatric comorbidity has been shown to influence disability outcomes in multiple sclerosis.^5^ Furthermore, one of the most prevalent psychiatric comorbidities, depression, plays a significant role in non-compliance with medical treatment,^6^ which makes the absence of multidisciplinary approaches all the more regrettable.

Publications are starting to emerge with the aim of testing quick and cost-effective screening procedures for neurological patients^7^,^8^ with a primary focus on depression and anxiety, the prevalence of which has been estimated at 34–39% in an Arabic cohort.^9^ Other studies confirm the heightened prevalence of depression and anxiety for various neurological illnesses.^10–13^ However, additional screening procedures could explore symptoms beyond anxiety and depression, as it is likely that some neurological illnesses will manifest with other signs of distress. For instance, neurodegenerative disorders such as dementia, Parkinson’s Disease, and multiple sclerosis are known to be associated with emotion dysregulation, impulsivity, aggression, obsessive-compulsive behaviors, paranoia or psychosis that are not commonly captured by screening instruments for depression or anxiety. In addition to showing a higher incidence rate of depression and anxiety, patients with multiple sclerosis are more frequently affected by bipolar disorders and schizophrenia.^13^ Moreover, the prevalence of comorbid disorders of patients with epilepsy has been shown to be significantly higher for depression, anxiety, psychotic and attention-deficit disorders compared to the general population.^3^

The importance of an intensive analysis of psychiatric symptoms in neurological diseases is further emphasized by the neural basis and associated changes of such diseases. Neurological diseases lead to cellular and structural changes in the brain, including inflammation, dysregulation of specific neural circuits and metabolic changes, and in the body, resulting in dysfunctional brain-body interactions (Di Gregorio and Battaglia, 2024).^14^ For example, dysmetabolism within the kynurenine pathway is associated with neuropsychiatric diseases such as MS, Parkinson’s and Alzheimer’s, and psychiatric diseases such as depression and schizophrenia.^15^,^16^ Neurodegeneration is also associated with both psychiatric and neurological diseases.^17^ In addition, brain regions associated with emotion dysregulation, such as the prefrontal cortex and amygdala,^18^,^19^ may also be affected by neurological disease (eg, dysfunction). Therefore, the mechanisms of neurological and psychiatric diseases share some common developmental underpinnings, highlighting the importance of further research.

Additional information would thus be helpful in providing neurologists with a standardized tool for screening for a wide range of symptoms and to facilitate a more comprehensive, multidisciplinary treatment, resulting in better outcomes and an efficient use of healthcare resources.

To this end, we screened a sample of 437 neurological inpatients with the German version of the Brief-Symptom-Inventory-53 (BSI-53),^20^,^21^ a short, self-report instrument designed to measure nine specific symptom dimensions associated with psychiatric disease, including somatization, obsessive-compulsion, interpersonal sensitivity, depression, anxiety, hostility, phobic anxiety, paranoid ideation, and psychoticism, as well as global measures of distress. We expected to find clinically relevant psychological distress even without the presence of depression and anxiety in our patients. Additionally, we administered the German version of the Hospital Anxiety and Depression Scale (HADS-D)^22^,^23^ in order to substantiate previous findings about the high prevalence of such symptomatology in neurological patients and to further examine the impact of age and gender on the frequency thereof. Due to the different comorbidities reported for different neurological diseases in previous literature, we investigated, whether we could find differences in mental distress between the neurological diseases. Furthermore, age has been reported to play a role in the effect life-limiting diseases on the prevalence of anxiety and depression^24^ and on the prevalence of psychological distress in the general population.^25^,^26^

### Goal of This Study

Our goal was to further highlight the need for psychiatric screening in neurological treatment and thus pave the way towards a multidisciplinary approach to disease management.^1^,^2^

## Methods

### Participants

835 consecutive patients admitted to the Department of Neurology without preexisting psychiatric main diagnoses were screened. 437 patients (188 males; 43.0%) filled in and returned the HADS-D and BSI-53 scales with a median age of 60.0 years (mean: 58.3; SD: 18.7; range: 18–96) belonging to five diagnostic groups based on ICD-10 criteria: vascular (n=128), demyelinating (n=80), degenerative (n=34), epileptic (n=48), other (n=133) with the group other including a wide variety of neurological disorders that are less common than the diagnoses of the other groups. Our sample, including preexisting secondary psychiatric illnesses, was chosen for reasons of comparability with the norm sample, because we assume the general population to be affected by psychiatric disorders in a comparable way,^27^ with only a low proportion of people being in professional treatment.^28^

### Procedure

Patients admitted to the Department of Neurology of the LVR-Clinic Düsseldorf between October 2015 and July 2016 were included in the study. After receiving ethical approval from the local ethical committee of the Medical Faculty of the Heinrich Heine University Düsseldorf, the data was retrospectively analyzed.

### Data Analysis

#### Initial Analyses

The 835 patients had a primary neurological diagnosis but no primary psychiatric diagnosis. Four further patients were excluded because they were younger than 18 years. After excluding all patients who did not return the HADS-D and the BSI-53 (N=398) 437 patients remained for the final analyses.

#### Statistical Tests

We used non-parametric chi-square tests and binary logistic regression for our analyses, which were all carried out using SPSS V.25. For multiple testing we used Bonferroni correction.

#### Outcome Measures

Overall distress: The BSI-53 (Brief Symptom Inventory, German version) is a short self-report symptom scale designed to measure nine specific symptom dimensions during the past seven days associated with psychiatric disease, including somatization, obsessive-compulsion, interpersonal sensitivity, depression, anxiety, hostility, phobic anxiety, paranoid ideation, and psychoticism, as well as global measures of distress.^21^ All items are assessed on scales ranging from 0 (not at all) to 4 (extremely), resulting in three global scores. The global scores are the Global Severity Index (GSI), Positive Symptom Distress Index and Positive Symptom Total. Franke^21^ reports an acceptable to high internal consistency (scale level > 0.70, global assessment > 0.90) and sufficient psychometric properties. In our utilization of the BSI-53, in addition to analyzing individual symptom scales, our focus was on the GSI as the most sensitive overall indicator of distress, combining information about the number of symptoms and their level. Raw scores were converted to T-values using the appropriate norm values for males and females in the German general population.^21^ According to the BSI-53 manual, a T-value of at least 63 in the GSI or in at least two of the nine subscales indicates a clinically relevant case that should receive further psychiatric testing and referral.^21^

Depression and anxiety: The HADS-D (German version of the Hospital Anxiety and Depression Scale,^22^ is a widely used assessment scale of symptoms associated with depression and anxiety. Fourteen items (seven per subscale) assess depressive and anxiety symptoms on a scale of 0 (low) to 3 (high) resulting in scores ranging from 0 to 21 for both depression and anxiety symptoms, with high scores indicating more severe symptoms. The HADS-D shows sufficient internal consistency α = 0.8 as well as psychometric properties.^29^ The HADS-D shows good sensitivity (SE) and specificity (SP) in neurological populations: epilepsy (for depression SE: 85.7%, SP: 80.2%),^30^ multiple sclerosis (for depression SE: 90%, SP: 87.3%; for anxiety SE: 73.2%, SP: 84.8%),^31^ Parkinson disease (for depression SE: 1, SP: 0.95).^32^

For the HADS-D we calculated separate scores for anxiety and depression (cut-off ≤8).^33^,^34^,

Age and gender: as age is known to correlate with symptom severity, we separated our sample into a younger and an older group by using a median split. Based on prior reports, we also took gender into consideration in our analyses.^7^ For the HADS, we compared our sample with the German norm sample.^35^ For comparisons between neurological diagnostic groups, we indicator coded the diagnostic groups with the group “other” as reference category. For supplemental regression analyses using the diagnostic group “vascular” as reference category see Tables S1–S4. In addition, the effects of the interaction terms of “diagnostic group and gender” as well as “diagnostic group and age” were separately tested for the main outcome parameters HADS and BSI-GSI (see Table S5).

## Results

### Anxiety Symptoms in Neurological Patients

In total, n= 119 patients (27.2%) had an HADS-D anxiety score above the cut-off. While we found no gender differences, young patients reached the cut-off for clinically relevant anxiety more often than older males and females, *X*^2^(1)=16.025, *p*<0.001 (see Table 1). According to the binary logistic regression with HADS-D anxiety scores as criterion and age category, gender and diagnostic group as predictors, a significant effect of age group on the HADS-D anxiety scores was found, but no other significant effects (see Table 2). In the additional analyses including different interaction terms “diagnostic group and gender” did not reach significance level (p=0.87) however “diagnostic group and age” evolve as significant (p=0.007; see Table S5).

**Table 1:** Patients [%(N)] Reaching the Cutoff of the HADS-D and the GSI of the BSI-53 Sorted by Gender, Age

	Male	Female
**All patients, HADS-D anxiety scale^a^**		
Young	36.4% (32)	35.3% (42)
Old	15.6% (15)	21.6% (26)
**All patients, HADS-D depression scale**		
Young	36.4% (32)	23.5% (28)
Old	21.9% (21)	24.2% (29)
**All patients, GSI score^b^**		
Young	53.4% (47)	50.4% (60)
Old	30.2% (29)	40.8% (49)
**Epilepsy patients, GSI score^c^**		
Young	66.6% (8)	62.5% (10)
Old	50.0% (2)	18.8% (3)
**Non-anxious/non-depressed patients, GSI score**		
Young	23.9% (11)	29.2% (21)
Old	16.4% (11)	21.3% (17)

**Notes**: Patients were split into the groups “Young” and “Old” by using a median split. ^a^significant differences between age groups (p<0.001) ^b^significant differences between age groups (p=0.005) ^c^significant differences between age groups (p=0.031).

**Table 2:** Binary Logistic Regression with HADS-D Anxiety Cut-off as Criterion and Gender, Age Group and Diagnostic Group as Predictors

Predictor	Odds Ratio	95%-CI [LL;UL]	Wald	*df*	*p*	Fit
(Intercept)	0.26		25.45	1	<0.001	
Gender	0.86	[0.55;1.34]	0.46	1	0.499	
Age Group^a^	2.51	[1.56;4.06]	14.21	1	<0.001	
Diagnostic Group^b^			1.94	4	0.746	
vascular (1)	1.11	[0.63;1.95]	0.14	1	0.713	
demyelinating (2)	0.78	[0.42;1.47]	0.59	1	0.444	
degenerative (3)	0.75	[0.28;2.00]	0.34	1	0.560	
epileptic (4)	0.75	[0.35;1.62]	0.54	1	0.465	
						*R*^2^ = 0.059

**Notes**: ^a^Age group is evaluated using the median. ^b^Indicator coded with the group “other” as indicator.

### Depressive Symptoms in Neurological Patients

In the HADS-D depression scale, n=111 patients (25.4%) showed a score beyond the cut-off. Again, younger males reached the cutoff more frequently than the older ones, however this tendency was not significant, *X*^2^(3)=6.348, *p*=0.096 (see Table 1). The binary logistic regression with HADS-D depression scores as criterion and age category, gender and diagnostic group as predictors found no significant effects either (see Table 3). Comparing our sample with an age-matched German norm population^35^ we found, after correcting for multiple comparisons, that no group reached the cut-off for depression more frequently than the normal population. As to the additional analyses including different interaction terms whether “diagnostic group and gender” nor “diagnostic group and age” did reach significance level (p=0.56 and p=40 respectively; see Table S5).

**Table 3:** Binary Logistic Regression with HADS-D Depression Cut-off as Criterion and Gender, Age Group and Diagnostic Group as Predictors

Predictor	Odds Ratio	95%-CI [LL;UL]	Wald	*df*	p	Fit
(Intercept)	0.24		27.75	1	<0.001	
Gender	1.33	[0.85;2.06]	1.58	1	0.209	
Age Group^a^	1.27	[0.79;2.03]	0.98	1	0.322	
Diagnostic Group^b^			2.15	4	0.708	
vascular (1)	1.15	[0.65;2.04]	0.24	1	0.625	
demyelinating (2)	1.30	[0.69;2.44]	0.65	1	0.420	
degenerative (3)	0.74	[0.28;2.00]	0.35	1	0.557	
epileptic (4)	1.47	[0.71;3.06]	1.06	1	0.303	
						R^2^ = 0.019

**Notes**: ^a^Age group is evaluated using the median. ^b^Indicator coded with the group “other” as indicator.

### Psychiatric Symptoms Beyond Anxiety and Depression in Neurological Patients

Overall n=133 patients (30.4%) showed distress as measured by the GSI score of the BSI-53 above the cut-off. Likewise, significantly higher rates of patients reaching the cut-off in the younger than in the older groups, *X*²(3)=13.057, *p*=0.005* (see Table 1), with a difference in the epileptic diagnostic group, *X*²(3)=8.886, *p*=0.031* (see Table 1). The binary logistic regression with the GSI Cut-Off as criterion and the predictors of age category, gender and diagnostic group revealed a significant effect for age category but not for the other predictors (see Table 4). Regarding the additional analyses including different interaction terms “diagnostic group and gender” did not reach significance level (p=0.31) however “diagnostic group and age” evolve again as significant (p=0.005; see Table S5). Given that our aim was to investigate symptoms beyond anxiety and depression, we first examined how many of our participants, who did not meet the cut-off for either depression or anxiety (HADS-D) (n = 274), would qualify as clinically relevant cases based on their results on other scales (BSI-53). This analysis showed that 23.9% of younger males, 29.2% of younger females, 16.4% of older males and 21.3% of older females reached the cut-off in at least two scales (BSI-Case), while their depression and anxiety levels were both in the normal range without an effect of diagnostic group (all *p*>0.256). A binary logistic regression with BSI-Case as criterion and the predictors of age category, gender and diagnostic group for patients that did not reach HADS-D depression and anxiety cutoffs revealed no significant effects after correcting for multiple testing (see Table 5).

**Table 4:** Binary Logistic Regression with GSI Cut-off as Criterion and Gender, Age Group and Diagnostic Group as Predictors

Predictor	Odds Ratio	95%-CI [LL;UL]	Wald	*df*	*p*	Fit
(Intercept)	0.33		19,43	1	<0.001	
Gender	0.92	[0.60;1.41]	0.17	1	0.684	
Age Group^a^	2.10	[1.33;3.31]	10.19	1	0.001	
Diagnostic Group^b^			4.26	4	0.372	
vascular (1)	0.96	[0.56;1.66]	0.02	1	0.883	
demyelinating (2)	0.95	[0.52;1.73]	0.03	1	0.858	
degenerative (3)	0.37	[0.12;1.14]	3.00	1	0.083	
epileptic (4)	1.32	[0.66;2.65]	0.62	1	0.327	
						*R*^2^ = 0.066

**Notes**: *95% CI, ^a^Age group is evaluated using the median. ^b^Indicator coded with the group “other” as indicator.

**Table 5:** Binary Logistic Regression for Patients That Did Not Reach HADS-D Cutoff for Depression or Anxiety with BSI-Case as Criterion and Gender, Age Group and Diagnostic Group as Predictors

Predictor	Odds Ratio	95%-CI [LL;UL]	Wald	*df*	*p*	Fit
(Intercept)	0.37		9.23	1	0.002	
Gender	0.73	[0.40;1.34]	1.04	1	0.308	
Age Group^a^	1.88	[1.00;3.55]	3.84	1	0.633	
Diagnostic Group^b^			8.02	4	0.091	
vascular (1)	0.77	[0.38;1.59]	0.49	1	0.484	
demyelinating (2)	0.26	[0.10;0.72]	6.82	1	0.009	
degenerative (3)	0.38	[0.10;1.43]	2.03	1	0.154	
epileptic (4)	0.68	[0.25;1.83]	0.58	1	0.445	
						*R*^2^ = 0.070

**Notes**: ^a^Age group is evaluated using the median. ^b^Indicator coded with the group “other” as indicator.

According to the different BSI-subscales (see Table 6) highest prevalence rates in specific symptom domains beyond “anxiety” (25.5%) and “depression” (20.6%) emerged in “somatization” (47.3%), “obsession-compulsion” (23.4%) and “hostility” (21.0%). Again, in none of the subscales differences between diagnostic groups evolved (all significance levels ≥.30).

**Table 6:** Prevalence [n (%)] of Mental Distress in Specific Symptom Domains According to BSI-53 Subscales (Standardized T-Score ≥63) by Diagnostic Groups

	Total (n=437)	Vascular (n=128)	Demyelinating (n=80)	Degenerative (n=34)	Epileptic (n=48)	Other (n=133)	p ^a^
Somatization	200 (47.3)	60 (46.9)	42 (52.5)	10 (29.4)	20 (41.7)	68 (51.1)	0.30
Obsession–compulsion	99 (23.4)	26 (20.3)	19 (23.8)	4 (11.8)	11 (22.9)	39 (29.3)	0.34
Interpersonal sensitivity	69 (16.3)	14 (10.9)	16 (20.0)	4 (11.8)	12 (25.0)	23 (17.3)	0.58
Anxiety	108 (25.5)	26 (20.3)	24 (30.0)	8 (23.5)	11 (22.9)	39 (29.3)	0.72
Depression	87 (20.6)	24 (18.8)	18 (22.5)	3 (8.8)	11 (22.9)	31 (23.3)	0.74
Hostility	89 (21.0)	24 (18.8)	19 (23.8)	2 (5.9)	13 (27.1)	31 (23.3)	0.56
Phobic anxiety	96 (22.7)	34 (26.6)	14 (17.5)	7 (20.6)	15 (31.3)	26 (19.5)	0.33
Paranoid ideation	81 (19.1)	22 (17.2)	19 (23.8)	4 (11.8)	13 (27.1)	23 (17.3)	0.72
Psychoticism	84 (19.9)	25 (19.5)	17 (21.3)	6 (17.6)	12 (25)	24 (18)	0.93

**Notes**: ^a^Significance level of “diagnostic group” in logistic regression (adjusted by age and gender).

In summary, about 25–30% of neuropsychiatric patients show mental distress exceeding the threshold for depression and anxiety, however also for other symptom domains like predominantly somatization or obsession-compulsion. Whereas no (significant) differences between diagnostic groups (vascular, demyelinating, degenerative, epileptic, other) evolved, risk for mental distress is significantly higher in younger (<60 years) patients.

## Discussion

Our results show that a large proportion of neurological patients exhibit clinically significant psychiatric distress, including a multitude of other psychiatric symptoms aside from depression and anxiety. Additionally, our findings corroborate previous reports of age being a relevant factor by showing that younger patients are most at risk.^7^,^36^ While we found no significant age or gender effects on depression, there was a non-significant tendency of young male patients more often reaching the cut-off for depression, also corroborating previous gender effects.^7^ Furthermore, results of the BSI-53 showed that symptoms beyond anxiety and depression also prevail among neurological patients. We also investigated whether different neurological diagnostic groups show a difference in the prevalence of psychiatric symptoms. However, after correcting for multiple testing we found no differences between diagnostic groups, indicating that the importance of screening for psychiatric disorders is not specific for certain neurological diagnoses. Likewise, we do not want to over-interpret the results of the additional analyses including interaction terms. Whereas the interaction of “diagnostic group and age” was (overall) highly significant regarding HADS-Anxiety and BSI-GSI, the specific comparisons indicate that mainly younger patients show a higher risk for mental symptoms compared to older patients across different diagnostic groups. Accordingly, this might rather be attributable to the significant (“main”) effect of age. Among the BSI subscales the scale “somatization” had the most widely reached cutoff, which might be explained by the real somatic problems experienced by patients with neurological diseases. In the subscales “obsession-compulsion” and “hostility” cutoffs were reached similarly often as depression and anxiety. This result exemplifies the importance of investigating psychiatric symptoms beyond depression and anxiety. Overall, while the previously described neuroanatomical and functional differences between the neurological diagnostic groups would imply differences in experienced psychiatric symptoms, such differences cannot be reported with sufficient certainty from the results of this study. This finding might indicate, that the relationship between the reported psychiatric symptoms and the neurological diseases might be less influenced by the neurofunctional changes related to neurological disease than by the overall effect such diseases have on life circumstances, especially for younger patients. However future studies should investigate underlying factors explaining the relationship between neurological and psychiatric comorbidities. Our results indicate, that psychiatric comorbidity is a widespread issue across a variety of neurological diseases. Taken together, our findings confirm that it is particularly important for neurologists to consider other psychiatric problems as well, especially in case of younger patients, and aim for a multidisciplinary treatment plan. Speculatively, the vulnerability of younger patients may be explained by the fact that these individuals find it harder to adjust to living with neurological problems than older ones, as these ailments represent a drastic impairment at ages when career productivity, financial security, family planning, and personal flexibility are of primary importance. The importance of age in the management of epilepsy has been previously shown by Escoffery et al.^37^ In their study they found that younger patients with epilepsy show less beneficial self-management behaviors than older patients. The development of behaviours that help dealing with a disease as epilepsy, might be one of the mechanisms why older patients with epilepsy showed less mental distress than younger patients. Which might be true for other neurological diseases as well. This would imply that a treatment of the mental distress would benefit from attaining improvements of self-management behavior. Furthermore the results of our study align with the results of a recent meta-analysis by Barker et al^24^ investigating the depression and anxiety in children, adolescents and young adults with life limiting medical diseases. The authors found a significantly heightened prevalence of both depression and anxiety for the clinical young adult group compared to the normal US young adult population. While a subgroup of patients with neurological diseases had a lesser occurrence of both psychiatric comorbidities compared to other medical life limiting diseases, the authors highlight the small number of patients with neurological diseases in their meta-analysis, further pointing to the need for future investigations of psychiatric comorbidities in young neurological patients. While the relationship between age and psychiatric comorbidities in the general population remains unclear, other researchers, as for example Kim et al^7^ found an increased occurrence of psychiatric comorbidities for young neurological patients as well. The authors discuss local economic development and management of psychiatric distress as possible explanations for the effect of age on the prevalence of psychiatric disorders. However, the effect of age on the prevalence of psychiatric symptoms in the general populations should also be considered as an explanation for our results, as in the past a higher prevalence of psychological distress has been found in younger individuals.^25^,^26^ Given the results of our study and the stated possible explanations for these results, the treatment plan for the younger individuals especially should be based on the cooperation of several disciplines such as psychology, psychiatry and neurology.

### Strengths

This investigation has a number of strengths. Though being a single-center study, our investigation acquired a large sample in a comparatively small amount of time by gathering the data as part of the routine treatment and diagnostic process. Thereby it serves as a cost-efficient example for future investigations of psychiatric comorbidities in clinical treatment of neurological diseases. Moreover, the methods we used provide an example for a change towards a multidisciplinary diagnostic and treatment process.

## Limitations

The current study provides insight into the high prevalence of psychiatric comorbidities going beyond depression and anxiety. The high dropout rate (about 48% non-returned questionnaires) and single-center design limit the study’s generalizability by introducing potential sample bias, reducing population diversity, and restricting the applicability of findings to broader contexts. Additionally, it should be noted, that the current investigation is of a screening nature. Future studies should investigate psychiatric comorbidities with psychiatric evaluations by trained clinicians to confirm the diagnoses indicated by the screening instruments. Furthermore, future studies could also compare self-report and clinician-rated measures as these measurements often show discrepancy regarding psychiatric evaluations.^38^ Moreover future prospective studies would strengthen the implications of the results found by our retrospective investigation. By including follow-up measurements and evaluations by professionals, the persistence and reliability of the reported symptoms could be further examined. Concerning the BSI, a limitation is found in the to our knowledge missing data for sensitivity and specificity as well as validity for populations with neurological diseases. Moreover, missing gender differences in mental distress, which have been reported for the BSI in the general population, might be related to the in-patient status, which might cause higher mental distress overall compared to outpatient-settings, causing minor differences between gender groups to diminish.

## Conclusions

In addition to highlighting the importance of psychiatric screening, our report also bears witness to the positive cost-benefit ratio of such a measure, considering the very little time and effort required for the completion of questionnaires and the significant value of the information gained.

## Data Availability

The datasets generated and/or analysed during the current study are not publicly available due to patient confidentiality but are available from the corresponding author on reasonable request (danbenz@hotmail.de). The deitentified shared data complies with relevant data protection and privacy regulations.

## References

[1] Hesdorffer DC. Comorbidity between neurological illness and psychiatric disorders. *CNS Spectrums*. 2016;21(3):230–238. doi:10.1017/S1092852915000929 26898322

[2] Dawood S, Poole N, Fung R, Agrawal N. Neurologists’ detection and recognition of mental disorder in a tertiary in-patient neurological unit. *BJPsych Bulletin*. 2018;42(1):19–23. doi:10.1192/bjb.2017.7 29388525 PMC6001869

[3] Kanner AM. When did neurologists and psychiatrists stop talking to each other? *Epilepsy Behav*. 2003;4(6):597–601. doi:10.1016/j.yebeh.2003.09.013 14698691

[4] Kanner AM. Management of psychiatric and neurological comorbidities in epilepsy. *Nat Rev Neurol*. 2016;12(2):106–116. doi:10.1038/nrneurol.2015.243 26782334

[5] McKay KA, Tremlett H, Fisk JD, et al. Psychiatric comorbidity is associated with disability progression in multiple sclerosis. *Neurology*. 2018;90(15):e1316–e1323. doi:10.1212/WNL.0000000000005302 29523642 PMC5894930

[6] DiMatteo MR, Lepper HS, Croghan TW. Depression is a risk factor for noncompliance with medical treatment: meta-analysis of the effects of anxiety and depression on patient adherence. *Arch Intern Med*. 2000;160(14):2101–2107. doi:10.1001/archinte.160.14.2101 10904452

[7] Kim J, Kim Y, Bae JS, Lee JH, Song HK. Concomitant psychiatric symptoms in neurological outpatients. *Int J Environ Res Public Health*. 2019;16(5). doi:10.3390/ijerph16050860 PMC6427503 30857273

[8] Jefferies K, Owino A, Rickards H, Agrawal N. Psychiatric disorders in inpatients on a neurology ward: estimate of prevalence and usefulness of screening questionnaires. *J Neurol Neurosurg*. 2007;78(4):414–416. doi:10.1136/jnnp.2006.103044 PMC2077768 17056626

[9] Alsaadi T, Kassie S, Mohamed Ali O, Mozahem K, Al Fardan S, Ahmed AM. Psychiatric comorbidity in neurological disorders: towards a multidisciplinary approach to illness management in the United Arab Emirates. *Front Psychiatry*. 2019;10:263. doi:10.3389/fpsyt.2019.00263 31073293 PMC6495369

[10] Tellez-Zenteno JF, Patten SB, Jetté N, Williams J, Wiebe S. Psychiatric comorbidity in epilepsy: a population-based analysis. *Epilepsia*. 2007;48(12):2336–2344. doi:10.1111/j.1528-1167.2007.01222.x 17662062

[11] Latas M, Vučinić Latas D, Spasić Stojaković M. Anxiety disorders and medical illness comorbidity and treatment implications. *Curr Opin Psychiatry*. 2019;32(5):429–434. doi:10.1097/YCO.0000000000000527 31116127

[12] Gaitatzis A, Trimble MR, Sander JW. The psychiatric comorbidity of epilepsy. *Acta Neurol Scand*. 2004;110(4):207–220. doi:10.1111/j.1600-0404.2004.00324.x 15355484

[13] Marrie RA, Fisk JD, Tremlett H, et al. CIHR team in the epidemiology and impact of comorbidity on multiple sclerosis. differences in the burden of psychiatric comorbidity in MS vs the general population. *Neurology*. 2015;85(22):1972–1979. doi:10.1212/WNL.0000000000002174 26519542 PMC4664123

[14] Di Gregorio F, Battaglia S. The intricate brain–body interaction in psychiatric and neurological diseases. *Adv Clin Exp Med*. 2024;33(4):321–326. doi:10.17219/acem/185689 38515256

[15] Battaglia MR, Di Fazio C, Battaglia S. Activated tryptophan-kynurenine metabolic system in the human brain is associated with learned fear. *Front Mol Neurosci*. 2023;16. doi:10.3389/fnmol.2023.1217090 PMC10416643 37575966

[16] Tanaka M, Szabó Á, Vécsei L. Redefining roles: a paradigm shift in tryptophan–kynurenine metabolism for innovative clinical applications. *Int J Mol Sci*. 2024;25(23):12767. doi:10.3390/ijms252312767 39684480 PMC11640972

[17] Battaglia S, Avenanti A, Vécsei L, Tanaka M. Neurodegeneration in cognitive impairment and mood disorders for experimental, clinical and translational neuropsychiatry. *Biomedicines*. 2024;12(3):574. doi:10.3390/biomedicines12030574 38540187 PMC10968650

[18] Fernandez KC, Jazaieri H, Gross JJ. Emotion regulation: a transdiagnostic perspective on a new RDoC domain. *Cognitive Ther Res*. 2016;40(3):426–440. doi:10.1007/s10608-016-9772-2 PMC4979607 27524846

[19] Schulze L, Schulze A, Renneberg B, Schmahl C, Niedtfeld I. Neural correlates of affective disturbances: a comparative meta-analysis of negative affect processing in borderline personality disorder, major depressive disorder, and posttraumatic stress disorder. *Biol Psychiatry Cogn Neurosci Neuroimag*. 2018;4(3):220–232. doi:10.1016/j.bpsc.2018.11.004 30581154

[20] Derogatis LR, Melisaratos N. The brief symptom inventory: an introductory report. *Psychol Med*. 1983;13(3):595–605. doi:10.1017/S0033291700048017 6622612

[21] Franke GH. *BSI Brief Symptom Inventory - Deutsche Version. Manual*. Göttingen: Beltz; 2000.

[22] Herrmann C, Buss U. *Vorstellung Und Validierung Einer Deutschen Version Der “Hospital Anxiety and Depression Scale”(HAD-Skala). Ein Fragebogen Zur Erfassung Des Psychischen Befindens Bei Patienten Mit Körperlichen Beschwerden*. Diagnostica; 1994.

[23] Zigmond AS, Snaith RP. The hospital anxiety and depression scale. *Acta Psychiatr Scand*. 1983;67(6):361–370. doi:10.1111/j.1600-0447.1983.tb09716.x 6880820

[24] Barker MM, Beresford B, Bland M, Fraser LK. Prevalence and incidence of anxiety and depression among children, adolescents, and young adults with life-limiting conditions: a systematic review and meta-analysis. *JAMA Pediatr*. 2019;173(9):835–844. doi:10.1001/jamapediatrics.2019.1712 31282938 PMC6618774

[25] Jorm AF, Windsor TD, Dear KBG, Anstey KJ, Christensen H, Rodgers B. Age group differences in psychological distress: the role of psychosocial risk factors that vary with age. *Psychol Med*. 2015;35(9):1253–1263. 10.1017/S0033291705004976 16168148

[26] Keyes KM, Nicholson R, Kinley J, et al. Age, period, and cohort effects in psychological distress in the United States and Canada. *Am J Epidemiol*. 2014;179(10):1216–1227. doi:10.1093/aje/kwu029 24692432 PMC4010185

[27] Jacobi F, Höfler M, Strehle J, et al. Psychische störungen in der allgemeinbevölkerung: studie zur gesundheit erwachsener in Deutschland und ihr zusatzmodul psychische gesundheit (DEGS1-MH) mental disorders in the general population: study on the health of adults in Germany and the additional module mental health (DEGS1-MH). *Der Nervenarzt*. 2014;85(1):77–87. doi:10.1007/s00115-013-3961-y 24441882

[28] Mack S, Jacobi F, Gerschler A, et al. Self-reported utilization of mental health services in the adult German population - evidence for unmet needs? Results of the DEGS1-mental health module (DEGS1-MH). *Int J Methods Psychiatr Res*. 2014;23(3):289–303. doi:10.1002/mpr.1438 24687693 PMC6878535

[29] Herrmann-Lingen C, Buss U, Snaith P. Hospital anxiety and depression scale - deutsche version: deutsche adaptation der hospital anxiety and depression scale (Hads) von RP snaith und as zigmond hogrefe. 2018.

[30] De Oliveira GN, Lessa JMK, Gonçalves AP, Portela EJ, Sander JW, Teixeira AL. Screening for depression in people with epilepsy: comparative study among Neurological Disorders Depression Inventory for Epilepsy (NDDI-E), Hospital Anxiety and Depression Scale Depression Subscale (Hads-D), and Beck Depression Inventory (BDI). *Epilepsy Behav*. 2014;34:50–54. doi:10.1016/j.yebeh.2014.03.003 24681386

[31] Honarmand K, Feinstein A. Validation of the hospital anxiety and depression scale for use with multiple sclerosis patients. *Mult Scler J*. 2009;15(12):1518–1524. doi:10.1177/1352458509347150 19965520

[32] Mondolo F, Jahanshahi M, Granà A, Biasutti E, Cacciatori E, Di Benedetto P. The validity of the hospital anxiety and depression scale and the geriatric depression scale in Parkinson’s disease. *Behav Neurol* 2006;17(2):109–115. doi:10.1155/2006/136945 16873922 PMC5471536

[33] Bjelland I, Dahl AA, Haug TT, Neckelmann D. The validity of the hospital anxiety and depression scale. *J Psychosom Res*. 2002;52(2):69–77. doi:10.1016/s0022-3999(01)00296-3 11832252

[34] Spinhoven P, Ormel J, Sloekers PP, Kempen GI, Speckens AE, van Hemert AM. A validation study of the Hospital Anxiety and Depression Scale (Hads) in different groups of Dutch subjects. *Psychol Med*. 1997;27(2):363–370. doi:10.1017/s0033291796004382 9089829

[35] Hinz A, Brähler E. Normative values for the Hospital Anxiety and Depression Scale (Hads) in the general German population. *J Psychosom Res*. 2011;71(2):74–78. doi:10.1016/j.jpsychores.2011.01.005 21767686

[36] Fink P, Hansen MS, Søndergaard L, Frydenberg M. Mental illness in new neurological patients. *J Neurol Neurosurg*. 2003;74(6):817–819. doi:10.1136/jnnp.74.6.817 PMC1738470 12754363

[37] Escoffery C, McGee RE, Bamps Y, Helmers SL. Differences in epilepsy self-management behaviors among young and older adults. *Austin J Neurol Disord Epilepsy*. 2016;3(1):1015.

[38] Seemüller F, Schennach R, Musil R, et al. A factor analytic comparison of three commonly used depression scales (HAMD, MADRS, BDI) in a large sample of depressed inpatients. *BMC Psychiatry*. 2023;23(1). doi:10.1186/s12888-023-05038-7 PMC10386606 37507656

